# Metabolic Profiles of Propofol and Fospropofol: Clinical and Forensic Interpretative Aspects

**DOI:** 10.1155/2018/6852857

**Published:** 2018-05-24

**Authors:** Ricardo Jorge Dinis-Oliveira

**Affiliations:** ^1^Institute of Research and Advanced Training in Health Sciences and Technologies (IINFACTS), Department of Sciences, University Institute of Health Sciences (IUCS), CESPU, CRL, Gandra, Portugal; ^2^UCIBIO-REQUIMTE, Laboratory of Toxicology, Department of Biological Sciences, Faculty of Pharmacy, University of Porto, Porto, Portugal; ^3^Department of Public Health, Forensic Sciences, and Medical Education, Faculty of Medicine, University of Porto, Porto, Portugal

## Abstract

Propofol is an intravenous short-acting anesthetic widely used to induce and maintain general anesthesia and to provide procedural sedation. The potential for propofol dependency and abuse has been recognized, and several cases of accidental overdose and suicide have emerged, mostly among the health professionals. Different studies have demonstrated an unpredictable interindividual variability of propofol pharmacokinetics and pharmacodynamics with forensic and clinical adverse relevant outcomes (e.g., pronounced respiratory and cardiac depression), namely, due to polymorphisms in the UDP-glucuronosyltransferase and cytochrome P450 isoforms and drugs administered concurrently. In this work the pharmacokinetics of propofol and fospropofol with particular focus on metabolic pathways is fully reviewed. It is concluded that knowing the metabolism of propofol may lead to the development of new clues to help further toxicological and clinical interpretations and to reduce serious adverse reactions such as respiratory failure, metabolic acidosis, rhabdomyolysis, cardiac bradyarrhythmias, hypotension and myocardial failure, anaphylaxis, hypertriglyceridemia, renal failure, hepatomegaly, hepatic steatosis, acute pancreatitis, abuse, and death. Particularly, further studies aiming to characterize polymorphic enzymes involved in the metabolic pathway, the development of additional routine forensic toxicological analysis, and the relatively new field of ‘‘omics” technology, namely, metabolomics, can offer more in explaining the unpredictable interindividual variability.

## 1. Introduction

A general anesthetic is an unrecognizable chemical drug class that depresses all excitable tissues and produces a reversible state of unconsciousness, with absence of pain sensation over the entire body [1]. The pattern of depression is irregular and descending, with higher cortical functions (i.e., conscious thought, memory, motor control, and perception of sensations) being firstly depressed comparatively to medulla, which contains cardiovascular and respiratory vital centers [1]. In contrast to local anesthetics, the general anesthetics exert their main effects on the central nervous system. It should also be noted that a drug may have useful anesthetic actions without being a good analgesic and* vice versa*.

Before the development of effective anesthetics and analgesics, as well as blood transfusions and antibiotics, successful major surgery was virtually impossible owing to the severe pain, hemorrhage, and infection; the patient was usually tied, held down, or rendered unconscious by hypoxia, concussion, or high doses of natural central nervous system depressants such as ethanol or opioids [2]. For a drug to be useful as a general anesthetic, its actions must be of rapid onset, effective during the duration of the surgical procedure, then rapidly reversible. Therefore, only those that have short half-lives and that can be continually administered are useful, which is not the case of ethanol, benzodiazepines, and the majority of barbiturates [2]. General anesthesia is usually induced by intravenous injection of anesthetic agent (e.g., ultrashort-acting barbiturates such as thiopental and nonbarbiturates such as propofol, ketamine, and etomidate) and then maintained by inhalation of a gas (e.g., nitrous oxide) mixed with the vapor of a volatile liquids (e.g., desflurane and sevoflurane) and oxygen [3]. Intravenous anesthetic agents are usually defined as drugs that induce loss of consciousness in one arm-brain circulation time (normally 10–20 s), when given in an appropriate dosage.

Propofol (2,6-diisopropylphenol; alkylphenol derivative) was firstly introduced into the market in 1977 by Kay and Rolly [4, 5] and is currently the most frequently administered anesthetic drug that largely replaced barbiturates as an induction agent due to its favorable side effects profile, namely, fewer incidences of nausea, vomiting, and postoperative drowsiness [2, 6–8]. Since its pharmacokinetic profile allows for continuous infusions, it is also used for maintenance of general anesthesia (Table 1) either as part of a “balanced anesthesia” regimen in combination with volatile anesthetics, nitrous oxide, sedative-hypnotics, neuromuscular blocking drugs, and opioids or as part of a total intravenous anesthetic technique, usually in combination with opioids. It is also used as hypnotic for patients undergoing mechanical ventilation and for conscious sedation, especially in day-surgery or noninvasive (e.g., radiation therapy, endoscopy, and magnetic resonance imaging) procedures as it causes less nausea and vomiting than do inhalation anesthetics, and it is devoid of analgesic properties. The empirical formula of propofol is C_12_H_18_O, with two isopropyl groups positioned on each side of a hydroxyl group in the* ortho*-position on a phenol ring.

Several studies have demonstrated an unpredictable interindividual variability of propofol pharmacokinetics and pharmacodynamics with forensic and clinical adverse relevant outcomes (e.g., pronounced respiratory, cardiac depression, and “propofol-related infusion syndrome” (PRIS)), namely, due to polymorphisms in the UDP-glucuronosyltransferase (UGT) and cytochrome P450 (CYP) isoforms and drugs administered concurrently [9–13]. Therefore, due to its narrow therapeutic interval, propofol should be administered only by individuals trained in airway management, and titration of the induction dose helps to prevent severe hemodynamic changes [14]. Moreover, propofol is a final metabolite of other anesthetics such as fospropofol.

The focus of this manuscript is the metabolism and metabolomics of propofol, which is not well characterized in most studies. In the last few years several advances have been made in the knowledge of the specific enzymes isoforms involved in its metabolism, genetic influence, or the pharmacological interactions with other drugs. This work aims to review the preclinical and clinical metabolism and metabolomics of propofol, pharmacological- and toxicological-related effects in special populations, and analytical considerations. Some important reviews were previously published but metabolism focused only on major metabolic routes and not combined in harmonized and complete pathways. This integrated overview can also be a useful support in evaluating the exact analytical needs and the most suitable techniques and procedures to obtain reliable and complete pharmacokinetic data [15–18].

## 2. Methodology

Aiming to obtain as much as complete integrative review, we carried out an English, German, French, Portuguese, and Spanish exhaustive literature search to identify and analyze relevant articles. Propofol and derivatives and related known metabolizing enzymes and metabolites were searched in books and in PubMed (US National Library of Medicine) without a limiting period. Furthermore, electronic copies of the full papers were obtained from the retrieved journal articles and books on propofol and related compounds (e.g., fospropofol), which were further reviewed for possible additional publications related to human and nonhuman* in vivo* and* in vitro* studies.

## 3. Propofol Pharmacodynamics

Compared to other intravenous anesthetics, propofol has a favorable pharmacological profile, namely [7], (i) rapid onset and distribution; (ii) rapid metabolism; (iii) very rapid recovery; (iv) limited cumulative effect that is useful for day-case surgery; (v) being an achiral compound; and (vi) having a low incidence of nausea and vomiting.

The propofol central nervous system depression is probably mediated through *γ*-aminobutyric acid (GABA) and glutamatergic N-methyl-D-aspartate (NMDA) receptors, as agonist and antagonist, respectively [30]. Particularly, as a GABAergic anesthetic, propofol modulates the action of GABA at GABA_A_ receptors either by prolonging inhibitory postsynaptic currents mediated by GABA_A_ receptors or by enhancing GABA release via presynaptic mechanisms [31–34]. As widely described, GABA_A_ receptors are ligand-gated chloride channels made up of five subunits (generally comprising two *α*, two *β*, and one *γ* or *δ* subunit) [35, 36]. Anesthetics can bind to hydrophobic pockets within different GABA_A_ receptor subunits increasing the chloride influx current causing neuronal hyperpolarization. Specific mutations of the amino acid sequence of the *α* subunit inhibit the actions of volatile anesthetics but not those of intravenous anesthetics, whereas mutations of the *β* subunit inhibit both volatile and intravenous anesthetics [37, 38]. This suggests that volatile anesthetics may bind at the interface between *α* and *β* subunits (analogous to benzodiazepines that bind at the interface between *α* and *γ*/*δ* subunits), whereas the intravenous anesthetics may bind only on the *β* subunits. A further level of complexity arises because there are different subtypes of each subunit. Different subunit compositions give rise to subtly different subtypes of GABA_A_ receptor and these may be involved in different aspects of anesthetic action.

The *α*_2_-adrenoreceptor system also seems to play an indirect role in the sedative effects of propofol [39].

## 4. Propofol Formulations

As with other intravenous anesthetic agents, propofol presents an interesting pharmaceutical formulation problem: ideally an IV anesthetic agent must be highly lipid-soluble to cross the blood-brain barrier and act into highly perfused lipid-rich tissues (i.e., brain, spinal cord), yet sufficiently water-soluble to be formulated as a solution that can be safely injected intravenously. Since it is almost insoluble in water at pH 7.0, propofol is formulated as oil-in-water emulsions (similar to milk) since it forms a 1% aqueous emulsion with 10% soyabean oil, 2.25% glycerol as a tonicity/stabilizing agent, 1.25% lecithin as emulsifier, the major component of the egg yolk phosphatide fraction, and sodium hydroxide to adjust the pH [2]. Hence, susceptible patients may experience allergic reactions, but there is no scientific evidence even for those with immunoglobulin E confirmed allergy to egg, soy, or peanut [40]. The resulting formulation is a slightly viscous, milky-white isotonic solution (commonly called the “milk of amnesia” by anesthetists) with a pH of 7–8.5 for intravenous injection or infusion. Typically a propofol formulation of 1% (10 mg/mL) is available, but in some countries a 2% concentration is accessible primarily for continuous infusion. Due to oil-in-water emulsion, administration can cause pain on injection [41, 42]. Moreover, since the pharmaceutical formulation is an excellent medium for bacterial growth (e.g.,* Escherichia coli *or* Candida albicans*), asepsis during administration and storage is necessary [43]. Although retardants of bacterial growth [e.g., ethylenediaminetetraacetic acid (0.05 mg/mL), metabisulfite (0.25 mg/mL), or benzyl alcohol (1 mg/mL)] are added to the formulations by different manufactures, it is recommended that the contents of a vial should be discarded within 6 hours after being open. Unopened vials should be stored at 22°C and are not light sensitive. The addition of metabisulfite in one of the formulations has raised concern regarding its use in patients with reactive airway disease (e.g., asthma) or sulfite allergies. If storage of propofol is necessary, the vial contents should be aspirated into a sterile syringe, capped with a needle or collected into a plain sterile vacutainer, and used as soon as possible. There are reports of sepsis developing in patients that have been exposed to contaminated propofol [44, 45].

## 5. Pharmacokinetics of Propofol

### 5.1. Absorption

Table 2 presents pharmacokinetic data regarding propofol. Previous reports concluded that propofol itself has little or no oral bioavailability, presumably due to first-pass hepatic metabolism of at least 80% of an lipid emulsion in animals [46, 47] and in humans [48, 49]. Moreover, propofol is ineffectively given by either intramuscular or subcutaneous routes and therefore it is restricted to intravenous administrations.

### 5.2. Distribution

After a single bolus or continuous infusion, propofol as other intravenous anesthetics, is best described by a three-compartment linear model [50, 51]:

- A rapidly equilibrating plasma compartment.
- A fast equilibrating compartment (with a distribution half-life of 1–8 minutes) between plasma and highly perfused organs such as the lung, liver, kidneys, and brain. Indeed, due to its high lipid solubility (octanol/water partition coefficient of 4300) [52], propofol easily crosses the blood-brain barrier resulting in a rapid onset (few seconds; also referred to as within one arm-brain circulation time) of anesthesia. The patients are asked to count backwards from 10 as the propofol is injected and they rarely reach 4 or 3.
- A slowly equilibrating deep compartment (with a distribution half-life of 30–70 minutes) between central nervous system and less perfused tissues such as skeletal muscle and adipose tissue. However, this is a rapid redistribution (half-life of 2–4 minutes) that together with a fast metabolism justifies the propofol short duration of effect of only 3–5 minutes and therefore a short recovery period, speed of awakening, and few hangover effects.

Due to its rapid clearance from the central compartment, the slow return of propofol from the deep compartment has little influence on the initial rapid decrease in propofol concentrations. Although the influence of obesity on propofol pharmacokinetics is not entirely clear, the greater the amount of body fat, the briefer the effect of a single IV dose and the greater the distribution volume. Generally, the blood distributes more to nonadipose than to adipose tissues, resulting in higher plasma drug concentrations in obese patients than those patients with less adipose mass. With prolonged administration or large doses, saturation of fat depots leads to prolonged drug action and delayed recovery as drug is slowly released back into the circulation to be eliminated. Consequently, patients administered IV anesthetic agents for short-stay procedures must be advised that they cannot drive or take public transport home and need a responsible person to care for them for 24 hours. Furthermore, propofol clearance increases because of the increased liver volume and liver blood flow associated with obesity (and increased cardiac output).

Propofol is also highly protein-bound (97–99%), namely, to albumin, and therefore patients with hypoalbuminemia may require a lower dose for anesthesia induction [53–57]. It is also possible that propofol competes with other drugs for the same albumin binding site. To prolong anesthesia, small boluses can be given as required or alternatively an infusion can be administered. Propofol also tightly binds to erythrocytes [58].

### 5.3. Metabolism of Propofol

Although most volatile anesthetics are excreted unchanged by the lungs, with less than 5% being metabolized by CYP enzymes primarily CYP2E1, intravenous anesthetics undergo extensive metabolism by the CYP enzymes or UGT prior to excretion in the kidney. The rate of metabolism varies with species, age, the physical condition of the animal, and the presence or absence of concurrently administered drugs [59].

Propofol undergoes rapid and extensive metabolism to water-soluble inactive metabolites. The liver is the major metabolic site, but extrahepatic clearance of propofol has also been suggested since systemic propofol clearance exceeds hepatic blood flow [60, 61]. Indeed, in patients undergoing liver transplantation, the amount of propofol metabolite excretion did not decrease during the anhepatic phases when the liver was excluded from the circulation [62–65]. The lungs are responsible for approximately 30% of the uptake and first-pass elimination after a bolus dose [66]. During a continuous infusion of propofol, there is a 20% to 30% decrease of the propofol concentration measured across the lung in humans and a higher concentration of the metabolite 2,6-diisopropyl-1,4-quinol on the arterial side of the circulation [67]. The human brain, kidney, and small intestine are also important organs for extrahepatic metabolism of propofol [61].

**Figure 1:**
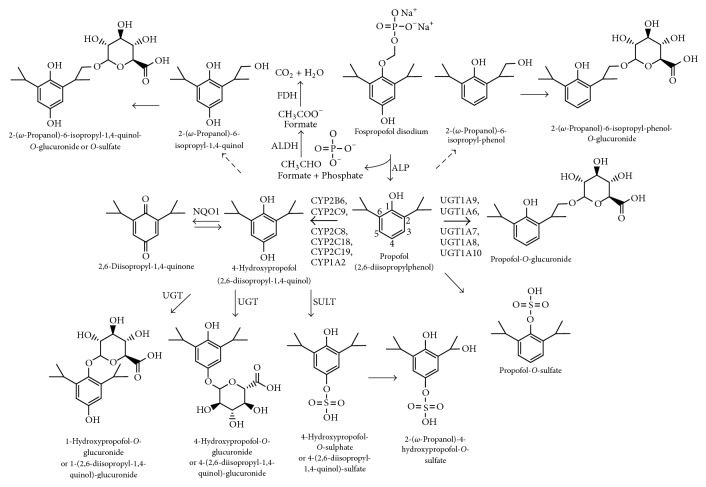
Metabolic pathway of propofol and fospropofol. Dashed arrows represent minor routes and both metabolites can undergo glucuronide and sulfate conjugation. SULT: sulfotransferase; UGT: UDP-glucuronosyltransferase; ALDH: aldehyde dehydrogenase; ALP: alkaline phosphatase; NQO1: diaphorase; CYP: cytochrome P450.

Metabolic pathways of propofol were studied in several species and include direct conjugation of the hydroxyl group and* p*-position aromatic or aliphatic hydroxylation followed by conjugation of 2,6-diisopropyl-1,4-quinol with glucuronic acid at C1 and C4 positions and sulfate at C4 position (Figure 1) [57, 60, 68, 69]. 2,6-Diisopropyl-1,4-quinol may undergo chemical or by-diaphorase (NAD(P)H dehydrogenase [quinone] 1; NQO1) conversion in 1,4-quinone by tautomeric equilibrium [70]. All metabolites are inactive, except 2,6-diisopropyl-1,4-quinol which has about one-third the hypnotic activity of propofol [71].

UGTs are phase II drug metabolizing enzymes that catalyze the glucuronidation of a wide variety of endobiotics and xenobiotics [9]. Propofol is primarily and extensively (~40% in humans) metabolized in liver, only through glucuronidation to propofol-glucuronide by enzymes coded by the UGT1A9 gene located on chromosome 2q37 [72]. UGT1A9 also conjugates other phenols, estrogen and thyroid hormones, acetaminophen, and SN-38 (an active metabolite of irinotecan) [73, 74]. The other main metabolites are the 1-glucuronide and the 4-glucuronide of the 1,4-quinol which account each for roughly 20% of the total propofol metabolites [75, 76]. UGT1A9 is primarily expressed in the liver but also in extrahepatic tissues such as kidney, small intestine, colon, and reproductive organs such as the testis and ovary [77, 78]. Variations in this gene significantly affect propofol metabolism; homozygous UGT1A9 726G/G variant has been associated with a loss of UGT1A9 activity [72], whereas the UGT1A D256N variant decreases propofol glucuronidation [9]. Authors suggested that carriers of D256N may be at risk of suffering adverse effects of propofol and other substrates that are primarily metabolized by UGT1A9 [9]. Mehlotra et al. found D256N with allele frequency 0.005 in Asian-Americans [79]. In a pilot study, Loryan et al. [10] found no association between the observed sex differences in propofol glucuronidation and UGT1A9 expression. The results were confirmed in a replication study [80]. However, patients who are heterozygous with UGT1A9-1887T/G variant required a statistically significant higher induction dose of propofol compared to those with other variants [81]. In addition, UGT1A9-133T/C was associated with higher propofol clearance (heterozygous with higher clearance than the rest). This study found no significant relationship between clinical differences and CYP2B6, GABRE, or other variants of the UGT1A9 gene [81]. A study of 150 Caucasian patients did not find any significant relationship between the four major haplotypes (set of DNA variants or polymorphisms that are inherited together) of the GABRE gene (that codes the GABA receptor, mRNA358G/T, 20118C/T, 20502A/T, and 20326C/T) and induction time with propofol [82].

The UGT1A6 has also been suggested to contribute to the hepatic and extrahepatic propofol metabolism [61, 83]. Although UGT1A9 is the main enzyme involved in the glucuronidation of propofol in humans, it does not exists in rats [84, 85]. However, UGT1A6 enzyme is detected in various tissues in rats and humans and has a similar homology as UGT1A9 [85, 86]. The UGT1A8, expressed mainly in the digestive organs but not in the liver, has also been shown to conjugate propofol [87, 88]. UGT1A7 and UGT1A10 also conjugate propofol in extrahepatic organs such as the kidneys and intestine [89].

*In vivo* studies previously identified marked species differences in the pharmacokinetic and metabolic profiles of propofol among rats, rabbits, and dogs [57, 68, 69], and these differences have been attributed to the levels of expression and function of the UGT isoform(s) responsible for propofol glucuronidation in each animal species and are sex-dependent [90].

Although with much less extent than glucuronidation, sulfation of propofol catalyzed by sulfotransferase (SULT) has also been described as minor metabolite involved in propofol clearance in humans [68, 91]. While the 1-OH group of propofol is sterically hindered by the two isopropyl groups and therefore coupling with the small SO_4_^−^ group would be much more likely than the addition of glucuronic acid, several studies demonstrated significantly higher concentrations of propofol-glucuronide compared to propofol sulfate [92, 93].

Regarding phase I metabolism, the propofol hydroxylation by CYP represents a minor metabolic route. Nevertheless, this route could be basis of metabolic interactions or relevant genetic polymorphisms [94]. Aromatic hydroxylation is partially mediated by CYP2C9 (~50%) or CYP2B6 in human liver, especially at low substrate concentration [95, 96]. Moreover, propofol was likely to be metabolized by additional isoforms such as CYP2A6, CYP2C8, CYP2C18, CYP2C19, and CYP1A2, especially when substrate concentrations are high [95]. From a clinical perspective, this low specificity among CYP isoforms may contribute to a low interindividual variability and reduced metabolic drug interactions [95], but opposite results have been observed [97]. Indeed, in spite of its low (1–5%) contribution to the total liver CYP content, CYP2B6 polymorphisms also have a significant impact on the CYP2B6-dependent metabolism of several clinically relevant drugs such as cyclophosphamide,* S*-methadone, efavirenz, nevirapine, bupropion, selegiline, and propofol [98, 99]. Propofol is predominantly* p*-hydroxylated by CYP2B6 into hydroxyl propofol [100]. The gene for CYP2B6 has become noteworthy as one of the most polymorphic CYP genes, with a 20- to 250-fold variation in interindividual CYP2B6 expression. CYP2B6 genetic variation is responsible for the interindividual variability in propofol metabolism [9, 82, 101]. Indeed, the occurrence of the CYP2B6*∗*6 allele has been associated with decreased enzyme activity compared to the wild type (CYP2B6*∗*1). Kansaku et al. [11] reported a high plasma concentration of propofol in individuals with CYP2B6 c.516G>T, a marker of the CYP2B6*∗*6 allele that correlated with awakening time and risk index score. The adverse reactions risk index score included age of >65 years and simultaneous occurrence of CYP2B6 cG516T and UGT1A9 1366C>T [11]. Thus, elderly patients with CYP2B6 c.G516T and UGT1A9 C1366T may be at a higher risk of propofol-related adverse events, such as PRIS and cardiovascular instability. In addition, of clinical relevance, women (particularly Hispanic-American women) express considerably higher hepatic levels of CYP2B6 protein than men. On average, women require high doses for induction, produce more metabolites, and recover more quickly than men during a propofol anesthetic [10, 80], which may lead to female subjects being at an increased risk of awareness during propofol anesthesia. Using a more sensitive measure of propofol concentration, Mastrogianni et al. [102] reported that women with the T allele in the CYP2B6 c.516G>T had a statistically significant higher plasma concentration of propofol than noncarriers. Other attempts to link CYP2B6 and propofol requirements of metabolism yielded no statistically significant results. Regarding the c.1075A>C polymorphism in the CYP2C9 genes did not affect the pharmacokinetic profile of propofol among Polish patients [97].

Introduction of a hydroxyl group on the isopropyl group (2-(*ω*-propanol)-6-isopropyl-phenol = 2-*ω*-phenol) and in both positions (2-(*ω*-propanol)-6-isopropyl-1,4-quinol = 2-*ω*-quinol) and glucuronidation and sulfation of these moieties was also described [103].

Previous investigations have shown that propofol decreased animal [104] or human [94] CYP activities* in vitro*, perhaps by interacting with the haem moiety. Indeed, propofol itself results in a concentration-dependent inhibition of CYP3A4 and may alter the metabolism of drugs dependent on this enzyme such as opioids [105]. Moreover, since propofol reduces hepatic blood flow, the clearance of other drugs metabolized in the liver may be reduced [106]. Propofol is also known for modulating UGT1A1 activity for 4-methylumbelliferone and estradiol 3-*β*-glucuronidation [107].

### 5.4. Elimination

Propofol is mainly eliminated (73% of the dose in 24 h and 88% in 120 h) by glomerular filtration (renal clearance of 120 ml/min) as water-soluble metabolites and/or bile [108, 109]. Less than 1% of propofol is excreted unchanged in urine, and clearance is reduced in renal failure; only 2% is excreted in feces up to 48 h after dose [109, 110]. In humans, the major metabolite in urine is the glucuronic acid conjugate of propofol, which accounts for 53–73% of the total metabolites, depending mainly on the administered dose of propofol [111]. Enterohepatic circulation of propofol-glucuronide was demonstrated in rat and to a lesser extent in dog and absent in rabbit [71]. Glucuronic acid conjugates at the C1 or C4 positions and the sulfate conjugates at the C4 position of 2,6-diisopropyl-1,4-quinol (the ring-hydroxylated derivative of propofol) are the other metabolites recovered in human urine [111]. The long terminal elimination half-life of 4–23 hours indicates a deep compartment with limited perfusion, which results in a slow return of propofol back to the central compartment [2, 112]. Plasma clearance is high (20–30 mg/kg/min) and exceeds hepatic blood flow, indicating the importance of extrahepatic metabolism [62, 113]. Although most of a single dose of propofol is excreted within 24 hours, excretion from deep tissues into the urine may take up to six days with minor metabolites resulting from hydroxylation on the isopropyl side chain being recovered [75, 103].

## 6. Fospropofol: A Prodrug of Propofol

Fospropofol (2,6-diisopropylphenoxymethyl phosphate disodium salt) is a recently developed water-soluble derivative that, in comparison to propofol lipid emulsion, is less painful on injection and has lower risk of bacterial contamination and hypertriglyceridemia [7]. It was licensed in 2008 by the Food and Drug Administration (FDA) and is formulated as a clear to slightly yellow aqueous solution containing 3.5% of fospropofol, monothioglycerol (0.25% w/v), and tromethamine (0.12% w/v). The pH of the solution is 8.2–9.0. It is a phosphono-*O*-methyl prodrug that has a methyl phosphate group substituted at the C1 hydroxyl group of the propofol molecule that is rapidly converted by endothelial cell surface alkaline phosphatases to propofol (the active metabolite), phosphate, and formaldehyde [114]. The formaldehyde is then metabolized by aldehyde dehydrogenase (ALDH) in the liver and in erythrocytes to formate, which is then safely metabolized by 10-formyltetrahydrofolate dehydrogenase to CO_2_ and H_2_O with tetrahydrofolate as coenzyme, similarly to the other available phosphate methyl prodrugs such as fosphenytoin [115, 116]. Clinical studies did not note serum toxic formaldehyde concentrations, which may ultimately lead to metabolic acidosis, loss of vision, and death [117, 118]. Indeed, physiological or fospropofol infusion-related formate concentrations have been measured in the range of 13 ± 7 *μ*g/mL [119], while formate concentrations in patients who died of methanol intoxication were reported to be at least 350 times higher in the range of 7–11 mg/mL [120].

The available fospropofol formulation is a sterile, aqueous, colorless, and clear solution that is supplied in a single dose vial at a concentration of 35 mg/mL [2]. Since it is a prodrug, pharmacokinetics is more complex (Table 2) than for propofol itself. Fospropofol follows a two-compartment model while as mentioned previously a three-compartment model has been used to describe the kinetics of propofol [115]. The pharmacodynamics profile is similar to that of propofol, but fospropofol disodium has a higher onset of action and recovery is prolonged since the prodrug must first be converted into an active metabolite. Although patients receiving fospropofol do not appear to experience the injection pain typical of propofol, a common adverse effect is the experience of paresthesia, often in the perianal region, which occurs in up to 74% of patients. The mechanism for this effect is unknown. Fospropofol is currently approved for sedation during monitored anesthesia care.

Interestingly, oral administration of the fospropofol provides appreciable propofol bioavailability in both animal and human volunteers in comparison to the negligible oral bioavailability of propofol [121]. While propofol availability derived from the prodrug is appreciable, the bioavailability of the prodrug itself is low, suggesting that propofol is liberated from prodrug before entering the central compartment [121]. Most common adverse events include paresthesia and pruritus, probably caused by the phosphate ester component released and less serious hypoxemia and hypotension than propofol [26]. Since the molecular weight of propofol is 178.27 g/mol and the molecular weight of fospropofol is 332.24, both drugs are not equipotent, with 1.86 mg of propofol disodium being molar equivalent to 1 mg of propofol [122]. Serum toxic phosphate levels are not reached due to an efficient kidney elimination. Renal elimination of fospropofol is negligible (<0.02%) [27].

## 7. Propofol Metabolism and Related Urine Discoloration

**Figure 2:**
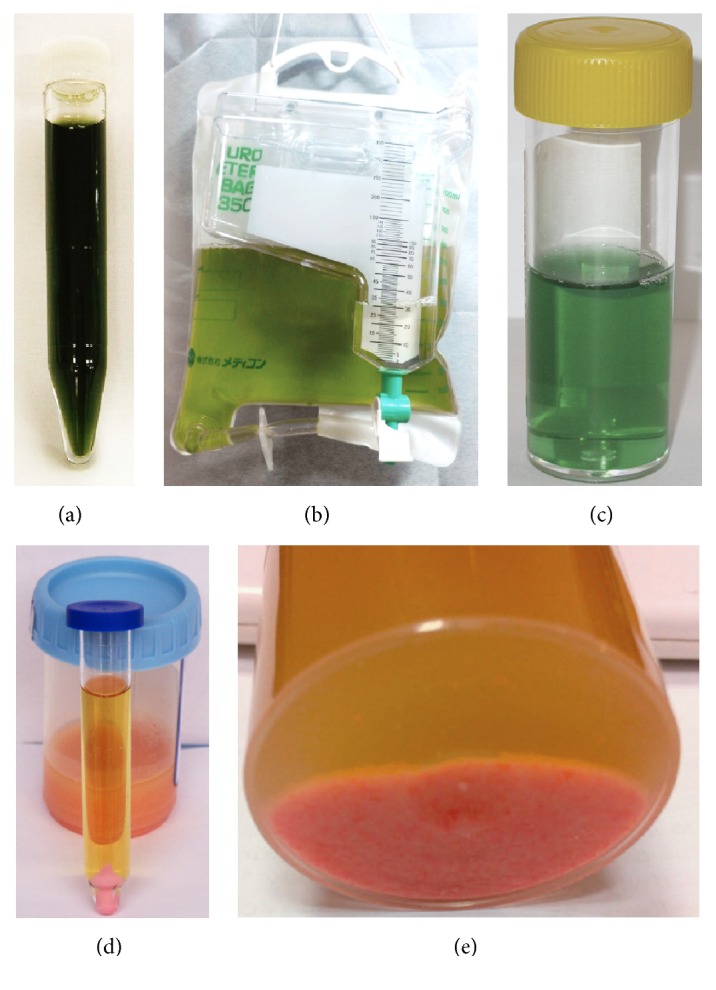
Green and pink urine discoloration after propofol infusion. Pink urine deposits/pellets were obtained after centrifugation. Reproduced with permission from (a) [19], (b) [20], (c) [21], (d) [20], and (e) [22].

Urine discoloration due to propofol infusion is a benign, nonnephrotoxic side effect that has been previously described several times in the literature; majority of cases are related to continuous and prolonged anesthesia and rarely seen in patients during induction of anesthesia or for sedation [123–128]. Case reports describe green, pink, white, brown, and red-brown urine associated with propofol use (Figure 2) [20, 129, 130]. Green discoloration represents majority of reported cases, but other compounds (both xenobiotics and endobiotics) such as biliverdin (in cases of long standing obstructive jaundice), amitriptyline, indomethacin, cimetidine, metoclopramide, methocarbamol, osmotic therapy and promethazine, methylene blue (bluish green), food coloring and indigo dyes, and urinary tract infection by* Pseudomonas aeruginosa* have also been implicated [131–136]. Since it is a nonharmful situation per se, neither healthcare staff nor relatives should be alarmed. The green urine usually resolved after propofol discontinuation in a matter of hours [137]. If urine analysis is normal, the accountable drug should be identified by pausing medications that are known for this side effect. The mechanism of propofol-induced urine green discoloration is unknown, but several causes have been proposed:

- Blakey and Hixson-Wallace [128] reported that the green discoloration of the urine due to propofol is attributable to the presence of phenolic chromophores resulting in hepatic metabolites of propofol, such as 1-glucuronide, 4-glucuronide, and 4-sulfate conjugates of 2,6-diisopropyl-1,4-quinol [109]. Although they are of no clinical significance, they do serve as an indicator of relative urine pH where alkalinization increases and acidification decreases formation of these phenolic derivatives [109]. Urinary discoloration can worsen at the time of alcohol consumption due to the augmented activity of CYP- and UGT-related clearance of propofol leading to higher concentration of its metabolites [138].
- Pedersen et al. [137] also reported that the discoloration appears when the clearance of propofol exceeds hepatic elimination and extrahepatic elimination of propofol occurs [139].
- Shioya et al. [19] reported that green urine was associated with enterohepatic circulatory failure due to constipation and impaired peristalsis, supplement of albumin and erythrocytes as carrier proteins due to administration of an albumin preparation and concentrated red cells, and extrahepatic glucuronidation in the kidneys.
- Fujii-Abe et al. [140] by performing liquid chromatography–mass spectrometry (LC-MS) registered 2 unique peaks in the green urine at 490 and 590 nm, with the complementary colors being orange and blue, respectively. It was assumed that green urine was observed because of a mixture of these 2 colors. Whereas the mass chromatographic peak corresponding to 490 nm was assumed to be L-urobilin, which is a common metabolite in normal urine, the peak corresponding to 590 nm was not identified. Moreover, the chromatographic peaks of the inactive metabolites 1-(2,6-diisopropyl-1,4-quinol)-glucuronide and 4-(2,6-diisopropyl-1,4-quinol)-sulfate were not observed in the urine samples, meaning that the real cause for urine discoloration remains unknown.

Besides urine, hair and liver green discoloration after propofol administration was also claimed to be due to phenol metabolites [141, 142]. Why this phenomenon is not observed in all propofol-treated patients is not known, but may, be partially related to prolonged administration. It is also widely reported that propofol is associated with transient pink urine discoloration possibly related to precipitation of amorphous uric acid crystals when urine osmolarity increases and pH is low [20, 22, 129, 143, 144]. Indeed, pure uric acid dihydrate crystals are colorless but can become pink when they absorb urinary pigments. The mechanism of pink urine remains unclear. It has been postulated that stress-related secretions of antidiuretics hormones increase the renal clearance of uric acid and cause uric acid crystalluria, which turns urine pink [145–147]. Indeed, obesity, especially with metabolic syndrome, may impair alkalinization of urine and thereby lead to the precipitation of uric acid. Moreover, surgery can stimulate antidiuretic hormone secretion. Stimulation of the V1 receptor can lead to impaired tubular resorption of uric acid, resulting in uric aciduria [20]. Pink urine is an unusual occurrence that has been described using some laxatives (phenolphthalein) or antipsychotics, such as chlorpromazine and thioridazine [148]. Full recovery without complications can be expected. Nevertheless, the risk of uric acid lithiasis should be borne in mind.

White urine discoloration was occasionally related to vehicle of the propofol emulsion [130].

## 8. Metabolomics of Propofol

The metabolomics of propofol was not yet extensively studied with only four publications reported in Medline. This is a relatively new field of ‘‘omics” technology that is primarily concerned with the ‘‘global” quantitative and qualitative biochemical characterization of small molecules (i.e., the metabolome) at specific time points, produced by the genome of the host organism and by the genomes of its microflora or deriving from food, drinks, pollutants, and drugs and their metabolites [15, 149]. It was recently evidenced that different anesthetics (i.e., propofol and etomidate) but at equipotent dose ranges induced small but meaningful differences in the plasma metabolic profiles by proton magnetic resonance spectroscopy (^1^HNMR) [150]. Moreover, Jacob et al. [151] found higher glucose and lactate levels with sevoflurane in the human brain compared with propofol. They suggest that findings could reflect greater neuronal activity with sevoflurane resulting in enhanced glutamate-neurotransmitter cycling, increased glycolysis, and lactate shuttling from astrocytes to neurons or mitochondrial dysfunction [151, 152]. Corroborating results, Alkire et al. [153] assessed cerebral glucose metabolism in volunteers by positron emission tomography (PET) before and during infusion of propofol to the point of unresponsiveness. The whole-brain metabolic rate decreased by 48% to 58%, with limited regional heterogeneity observed.

## 9. Propofol Abuse

Propofol abuse was firstly described in 1992 [154, 155] but only gained considerable attention in recent years mostly due to fatal prescription cases by celebrities (e.g., Heath Ledger and Michael Jackson) and by healthcare professionals [156, 157]. Indeed, since propofol has prescription status and is generally only distributed to anesthesiologist or physician's offices which commonly perform out-patient surgeries, it predisposes the healthcare specialist population to abuse [3]. Therefore, propofol is most commonly abused by nurses and physicians, especially those working frequently with anesthetics, but has recently also spread to wider populations as well [158, 159]. The abuse potential of propofol is generally regarded as low, namely, due to the short duration of action. Nevertheless, the fast onset of creating a short ecstatic and euphoric feeling, the capacity to induce sexual delusions, fantasies, and disinhibition, without many of the side effects that are associated with other drugs, and the fact of not being a controlled substance are the main reasons for its widespread abuse [158, 160]. Pharmacodynamically, propofol increases dopamine concentrations in the* nucleus accumbens*, a phenomenon noted with other addictive psychoactive substances [161–164]. Chronic propofol abuse can result in tolerance, and repeated injections exceeding 100 times per day have been reported [163, 165]. No physical dependence has been described by abusers and there are only scarce reports on possible withdrawal phenomena after the use of propofol for medical purposes [166, 167].

## 10. Adverse Effects, Fatal Intoxications, and Autopsy Findings

Table 1 presents major adverse and side effects of propofol. The pronounced respiratory and cardiac depression are relevant adverse outcomes. There have been also reports of a PRIS occurring in approximately 1 in 300 patients when it has been given in high doses and for a prolonged period to maintain sedation, particularly critically ill patients in intensive care units and children [168, 169]. PRIS is characterized by severe metabolic acidosis, skeletal muscle necrosis (rhabdomyolysis), hyperkaliemia, lipaemia, hepatomegaly, renal failure, arrhythmia and cardiovascular failure, and death. The pathophysiology of PRIS appears to involve a disturbance of mitochondrial metabolism by affecting *β*-oxidation of free fatty acids [168, 170, 171].

The risk of death due to self-administered propofol has been debated and several authors reported it to be low or absent, namely, due to the low concentration found in commercial ampoules (20 ml contains 200 mg propofol) that is equivalent to a standard dose of 2–2.5 mg/kg body weight to a healthy 80 kg individual to induce general anesthesia within 1-2 min after injection and arousal after 5–10 min [159, 172, 173]. Due to the fast-acting narcotic effect of propofol, the self-injection of more than one ampoule at a time is unlikely. However, victim can mix the content of one or more vials for rapid and continuous intravenous infusion and therefore administration will continue despite loss of consciousness [3]. Another challenge that faces forensic toxicologists is the fact that in most case reports of fatal propofol abuse, blood concentrations were lower or within the commonly accepted therapeutic range (1–8 *µ*g/mL) after a standard anesthetic induction dose [158, 159, 173, 174]. According to the short half-life, this may suggest that after losing consciousness, the victim probably survived enough time to reduce blood propofol level through distribution, metabolism, and excretion [175]. Nevertheless, for results interpretation it is important to remember that therapeutic levels of propofol apply to an anesthetized patient with respiratory support which is lacking in reported propofol abuse cases and that there is a wide variability of propofol plasma concentrations [173]. Moreover, propofol is also coabused with other drugs, namely, benzodiazepines, z-drugs, barbiturates, and opioids [158]. Indeed, a synergistic interaction has been found with benzodiazepines at the GABA_A_ receptors, such that the dose of propofol required to induce anesthesia should be reduced in the presence of midazolam [176].

One of the major drawbacks for the diagnosis of propofol forensic intoxications is the fact it is usually not included in standard drug screening analysis and, due to its low molecular weight and volatility, might be missed even in confirmatory exams. Although newer techniques are available for the direct detection of minute quantities of propofol in exhaled air of anesthetized patients [177], such approaches are not readily available in forensic institutions and therefore there is also a long field of opportunities to uncover abusive cases.

Autopsy and histological findings observed in lethal cases resulting from propofol overdose usually report cerebral and pulmonary edema, polyvisceral congestion, lungs with some petechial hemorrhages on pleural surface, hemorrhagic pancreatitis, and hepatic steatosis [158, 160, 178]. Although claimed as very rare idiosyncratic reaction (<1/10,000) and with a an estimated incidence of 0.1%–2% of all pancreatitis cases, hypertriglyceridaemia has been suggested as a causal relationship between propofol and pancreatitis since it is formulated as a fat emulsion [179]. Therefore, patients who develop hypertriglyceridaemia are at risk of developing pancreatitis, and serum triglyceride concentrations should be routinely monitored in these patients and alternative sedation strategies should be considered when hypertriglyceridemia is detected [178, 179]. Nevertheless, some case reports describe the development of propofol-induced acute pancreatitis in the absence of hypertriglyceridaemia [180].

## 11. Conclusion and Future Perspectives

Drugs with actions on the central nervous system are of particular importance in pharmacology, and major groups include anxiolytics, sedatives and hypnotics, antiepileptic, antipsychotics, antidepressant, antiparkinson, stimulants, general anesthetics, opioids, drugs for preventing or treating migraine, and miscellaneous drugs, including anticholinesterases, appetite suppressants, and centrally acting muscle relaxants. Other drugs may be administered to prevent or treat general pathologies to brain tissue (e.g., cytotoxic agents for tumors, antibiotics for infections, or anti-inflammatory agents in cerebral edema). Additionally, many drugs given for peripheral effects may cross the blood-brain barrier and have side effects on the central nervous system (e.g., autonomic drugs, antihistamines, and local anesthetics) and psychoactive illicit substances also exert central nervous system actions.

In this work, metabolism of propofol and respective genetic variability was fully reviewed. In humans, propofol produces inactive metabolites. It undergoes direct polymorphic* O*-glucuronidation in humans to propofol-glucuronide and hydroxylation to 2,6-diisopropyl-1,4-quinol. The latter substance subsequently undergoes phase II metabolism, resulting in the formation of further metabolites 4-(2,6-diisopropyl-1,4-quinol)-sulfate, 1- and 4-(2,6-diisopropyl-1,4)-glucuronides, or sulfates [103]. Further minor phase I propofol metabolites such as 2-(*ω*-propanol)-6-isopropylphenol and 2-(*ω*-propanol)-6-isopropyl-1,4-quinol are also described. Propofol is excreted in the urine after glucuroconjugation of the parent drug (to form the propofol-glucuronide) and sulfo- and glucuroconjugation of the hydroxylated metabolite to form 4-(2,6-diisopropyl-1,4-quinol)-sulfate, 1- or 4-(2,6-diisopropyl-1,4)-glucuronide, respectively. Current evidence suggests that close monitoring is required when administering anesthetics to individuals with the CYP2B6*∗*6 allele [181]. Nevertheless, additional studies are needed to elucidate and characterize polymorphic enzymes in explaining interindividual variations of the glucuronidation metabolic pathway and their pharmacological and toxicological adverse reactions. Although positive pharmacogenetic polymorphic associations have been found with clinical significance, the lack of reproducibility is a limitation, since most studies focus on single variant associations, while interindividual differences in propofol metabolism may be best explained through the contribution of multiple pathways. Indeed, the narrow therapeutic index and significant variability in patients' responses to anesthesia and surgery make the potential for severe adverse reactions high during the perioperative period. The identification of additional metabolites is also required to confirm xenobiotic exposure in a wider detection window, especially in alternative samples. Moreover, despite the fact that there are sex and racial/ethnic differences in response to propofol, to date, there is no strong evidence linking genetic variation to such observations, possibly due to the additional influence of weight, height, and lean body mass, environmental factors, and severe hepatic or renal impairment propofol pharmacokinetics [182–185]. Equally important is the potential for variation at the site of propofol action.* In vitro* it was shown that Y444W variant attenuates the effect of propofol, but associations with GABA_A_ receptor polymorphisms and clinical relevant effects of propofol need further studies [186]. Sites on the *β*_1_-subunit (M 286), *β*_2_-subunit (M 286), and *β*_3_-subunit (N265) of the transmembrane domains are crucial for the hypnotic action of propofol [187, 188]. The *α*-subunit and *γ*_2_-subunit subtypes also seem to contribute to modulating the effects of propofol on the GABA receptor [189].

Finally, metabolomics of propofol was not yet extensively studied and further studies are needed to clarify whether the different metabolomic patterns are significant from the clinical point of view, namely, taking into account sex, age, genetic polymorphisms, and different pathologic conditions.

## Figures and Tables

**Table 1:** Systemic therapeutic and adverse or side effects of propofol according to [2, 23–25].

Central Nervous System	
(i) Depression with decrease in cerebral blood flow (CBF) and cerebral metabolic rate for oxygen (CMRO_2_) and intracranial pressure (ICP), which may be important in a patient with raised ICP (head trauma, cerebral neoplasia) (ii) Decrease of intraocular pressure (useful in glaucoma) (iii) In comparison to thiopental is more or at least equipotent as anticonvulsant in the treatment of epilepsy (iv) Excitatory phenomena, including muscle twitching and rigidity, paddling and opisthotonus, are occasionally seen at induction. These reactions are believed to be subcortical in origin and are usually transient and proper treatment is rarely required	

Respiratory System	

(i) Respiratory depression (including apnea) after an induction dose is the most common side effect and may affect the fetus or neonate if used on pregnant women (category C) (ii) Decrease both respiratory rate and tidal volume, resulting in a rise in arterial partial pressure of carbon dioxide (hypercapnia) and acidosis (iii) Hypoxia is also possible in the patient breathing room air (iv) Bronchospasm in patients with reactive airway disease	

Cardiovascular System	

(i) Greater decrease of blood pressure than other injectable anesthetics due to decreases in myocardial contractility and systemic vascular resistance without a compensatory rise in heart rate. It is thought that it impairs the baroreceptor response to low blood pressure (ii) Depression that may lead to hypotension and bradycardia due to vasodilatation (iii) Hypertriglyceridemia (>500 mg/dL) when infused for greater than 72 hours since lipid emulsion contains 0.1 g of fat/mL (iv) Phlebitis and thrombosis	

Skeletal Muscle	

(i) Myoclonus is occasionally seen but does not trigger malignant hyperthermia	

Miscellaneous Effects	

(i) Antimuscarinic or atropine-like syndrome (i.e., agitation, tachycardia, confusion, hallucinations) that can be reversed by physostigmine (ii) Pancreatitis (iii) Allergic reactions have not been reported (iv) Pain on intravenous injection site is common, a fact that can be minimized by using a large vein and by injecting a local anesthetic (e.g., lidocaine) (v) No significant endocrine effect, no change in the coagulation profile or platelet count and no significant effect on gastrointestinal motility (vi) May have antioxidant effects similar to vitamin E (vii) Decrease nausea and vomiting (viii) Propofol infusion syndrome (ix) Pruritus (x) Benign urine discoloration (xi) Reduce lymphocyte proliferation and inhibits phagocytosis *in vitro* (xii) Abuse	

Precautions and Contraindications	

(i) Elderly, hypovolemic and hypotensive patients (ii) Benzodiazepines and other central nervous depressants	

**Table 2:** Comparison of propofol and fospropofol characteristics according to [14, 26–29].

Characteristics	Propofol	Fospropofol
Standard dose to induce general anesthesia	1.5–2.5 mg/kg (lipid emulsion formulation)	6.5 mg/kg (aqueous formulation)
Onset of action	40 s–1 min (“one arm-brain circulation”)	4–8 min
Duration of action after bolus dose	3–10 min	5–18 min
Volume of distribution (L/kg)	5.8	0.3
Total body clearance (L/h/kg)	3.2	0.36
Terminal phase elimination half-life (h)	0.97	0.88
Protein binding (%), mostly albumin	97–99	95–97
Side effect	Pain on injection and moderate to severe cardiovascular and respiratory depression	Transient perineal paresthesia, pruritus and mild cardiovascular and respiratory depression
